# Association between Maternal Fish Consumption and Gestational Weight Gain: Influence of Molecular Genetic Predisposition to Obesity

**DOI:** 10.1371/journal.pone.0150105

**Published:** 2016-03-01

**Authors:** Sofus C. Larsen, Lars Ängquist, Charles Laurin, Camilla S. Morgen, Marianne U. Jakobsen, Lavinia Paternoster, George Davey Smith, Sjurdur F. Olsen, Thorkild I. A. Sørensen, Ellen A. Nohr

**Affiliations:** 1 Research Unit for Dietary Studies at the Parker Institute, Bispebjerg and Frederiksberg Hospitals, the Capital Region, Copenhagen, Denmark; 2 Institute of Preventive Medicine, Bispebjerg and Frederiksberg Hospitals, The Capital Region, Copenhagen, Denmark; 3 MRC Integrative Epidemiology Unit, School of Social & Community Medicine, University of Bristol, Bristol, United Kingdom; 4 Department of Public Health, Section for Epidemiology, Aarhus University, Denmark; 5 Department of Epidemiology Research, Centre for Fetal Programming, Statens Serum Institut, 2300 Copenhagen S, Denmark; 6 Department of Nutrition, Harvard T. H. Chan School of Public Health, 655 Huntington Avenue, Boston, Massachusetts, United States of America; 7 Novo Nordisk Foundation Center for Basic Metabolic Research and Department of Public Health, Faculty of Health and Medical Sciences, University of Copenhagen, Copenhagen, Denmark; 8 Research Unit of Gynaecology and Obstetrics, Institute of Clinical Research, University of Southern Denmark, Odense, Denmark; University of Insubria, ITALY

## Abstract

**Background:**

Studies suggest that fish consumption can restrict weight gain. However, little is known about how fish consumption affects gestational weight gain (GWG), and whether this relationship depends on genetic makeup.

**Objective:**

To examine the association between fish consumption and GWG, and whether this relationship is dependent on molecular genetic predisposition to obesity.

**Design:**

A nested case-cohort study based on the Danish National Birth Cohort (DNBC) sampling the most obese women (n = 990) and a random sample of the remaining participants (n = 1,128). Replication of statistically significant findings was attempted in the Avon Longitudinal Study of Parents and Children (ALSPAC) (n = 4,841). We included 32 body mass index (BMI) associated single nucleotide polymorphisms (SNPs) and 5 SNPs found associated with GWG. BMI associated SNPs were combined in a genetic risk score (GRS). Associations between consumption of fish, GRS or individual variants and GWG were analysed, and interactions between fish and the GRS or individual variants were examined.

**Results:**

In the DNBC, each portion/week (150 g) of fatty fish was associated with a higher GWG of 0.58 kg (95% CI: 0.16, 0.99, P<0.01). For total fish and lean fish, similar patterns were observed, but these associations were not statistically significant. We found no association between GRS and GWG, and no interactions between GRS and dietary fish on GWG. However, we found an interaction between the *PPARG* Pro12Ala variant and dietary fish. Each additional Pro12Ala G-allele was associated with a GWG of -0.83 kg (95% CI: -1.29, -0.37, P<0.01) per portion/week of dietary fish, with the same pattern for both lean and fatty fish. In ALSPAC, we were unable to replicate these findings.

**Conclusion:**

We found no consistent evidence of association between fish consumption and GWG, and our results indicate that the association between dietary fish and GWG has little or no dependency on GRS or individual SNPs.

## Introduction

Many women gain excess weight during pregnancy, increasing the risk of complications during pregnancy and delivery, and in the longer term increasing the risk of both the mother and child of becoming overweight [1].

A woman’s dietary intake is a seemingly obvious prerequisite for excess gestational weight gain (GWG). In addition to total energy intake, the composition of nutrients and specific foods may also play a role [2]. Studies of diet among pregnant women are sparse, but results from a randomized controlled trial of 324 men and non-pregnant women suggest that increasing fish intake in an energy-restrictive diet can promote weight loss [3]. On the other hand, it has been suggested that a higher intake of fish or n-3 fatty acids during pregnancy may increase fetal growth rate [4], which could potentially cause a higher GWG. Results from the few published studies investigating the relationship between intake of fish and GWG have been inconsistent. Restall et al. (2014) found that a high intake of fish and seafood (defined as ≥3 servings week) was associated with a higher risk of excessive GWG among 1,950 women [5], while Merkx et al. (2015) found no relationship between a high consumption of fish (defined as ≥2 servings per week) and risk of excessive GWG among 455 women [6].

Several mechanisms, by which dietary fish could affect GWG, have been proposed. It has been suggested that n-3 fatty acids may decrease fat mass among non-pregnant [7;8], possibly by increasing fatty acid oxidation [8–10]. Furthermore, several studies in non-pregnant populations have found that high protein intake can cause weight loss, most likely by increasing satiety and thermogenesis [11]. Protein from fish may have a particularly beneficial effect on weight loss [3;12], perhaps due to a high content of the amino acid taurine [13–15]. However, in relation to GWG, this is complicated by the fact that these nutrients may also be associated with a higher growth in the foetus during pregnancy [16].

The degree of GWG may also to some extent be attributed to genetic factors, as indicated by a recent twin study [17], but knowledge of the involved variants is sparse. However, genome wide association studies (GWAS) have identified several genetic variants associated with higher BMI among non-pregnant woman and men [18], and although replication is lacking, some variants have also been suggested as candidates specifically for GWG [19]. Finally, a number of recently published studies have suggested that different dietary factors may modify the impact these genetic variants have on adiposity [20–22], but these studies were all conducted in non-pregnant populations.

The aim of our study was to examine the association between maternal fish intake (total, lean and fatty fish) and GWG, and explore whether this relationship was modified by genetic predisposition to higher BMI or GWG. For this purpose, we used the Danish National Birth Cohort (DNBC) as an exploratory cohort and the Avon Longitudinal Study of Parents and Children (ALSPAC) as a replication cohort.

## Materials and Methods

### The exploratory cohort

The DNBC enrolled a total 100,418 pregnancies among a total of 92,274 women during the years 1996 to 2002. The cohort is described in detail elsewhere [23;24]. Briefly, the women were recruited from all over Denmark at the beginning of pregnancy during their first antenatal visit to their general practitioner. Two computer-assisted telephone interviews were conducted during pregnancy at approximately week 16 and week 30 (Interviews 1 and 2), and twice after pregnancy at approximately 6 months and 18 months post-partum (Interviews 3 and 4). A self-administered food-frequency questionnaire (FFQ) was further mailed to the women around gestational week 25, to which 70,183 women responded. The FFQ was developed from the one used and validated in the Danish Diet, Cancer and Health Study [25–27]. All participants in DNBC provided written informed consent. The Scientific Ethic Committee in Denmark, the Danish Data Protection Agency, and the DNBC Steering Committee approved the study.

#### Sampling strategy

A nested case-cohort study was performed within the DNBC [28–30]. Women were only included if they had a blood sample taken during pregnancy and successful buffy coat extraction, had given birth to a live-born singleton, participated in Interview 1, and had given information about pre-pregnancy BMI (n = 67,853).

A total of 3,6% women with the largest residuals from the regression of BMI on age and parity (all included as continuous variables) were selected, as was a random sample of similar size from the remaining cohort. This gave a sample of 2,449 severely obese women (mean BMI 36.9) and 2,450 randomly selected women from the remaining cohort (mean BMI 23.1).

For the present study, further exclusions were made if information was missing on GWG (n = 1,363), if participants had a reported total energy intake below 2,500 kJ/day or above 25,000 kJ/day (n = 34), missing genetic data (n = 697), and missing dietary information (n = 687). After these exclusions, we had information on the selected genetic variants, dietary fish and GWG on a total of 2,118 women (990 obese and 1,128 from the random sample [non-obese]). A flow chart illustrating the selection of participants can be found in **S1 Fig.**

### The replication cohort

The replication cohort (ALSPAC) is a prospective population-based birth cohort that recruited 14,541 pregnant women resident in Avon, United Kingdom, with expected date of delivery between 1 April 1991 and 31 December 1992. The cohort is described in detail elsewhere [31]. The study was originally designed to assess the relationship between different environmental factors during pregnancy and the health or wellbeing of the child [31]. Ethical approval for the study was obtained from the ALSPAC Ethics and Law Committee and the Local Research Ethics Committees.

For the present study, we excluded women who had multiple births (n = 484), women who lost weight during pregnancy (n = 55) and women with missing total energy intake or recorded values below 2,500 kJ/day or above 25,000 kJ/day (n = 3,119). This gave a sample of 11,787 women. Further exclusions were made if information on GWG was missing (n = 847), if genetic data were missing (n = 4,017), or if information on covariates (BMI, socio-economic position, parity, etc) was missing (n = 2,082). After these exclusions, we had information on dietary fish, the necessary genetic information, GWG and covariates on a total of 4,841 women. Please note that the study website contains details of all the data that is available through a fully searchable data dictionary http://www.bris.ac.uk/alspac/researchers/data-access/data-dictionary.

To mimic the design from the DNBC sample, participants were categorized as obese (BMI >30) and non-obese (the remaining participants), ending up with a total of 238 obese participants and 4,603 non-obese participants. A flow chart illustrating the selection of participants can be found in **S2 Fig.**

### Gestational weight gain

In the DNBC, GWG was assessed, based on the question “How much (in kg) was your total weight gain in pregnancy?” posed in an interview 6 months post-partum.

In ALSPAC, six trained midwives abstracted data from obstetric medical records. The data abstractions included every measurement of weight entered into the medical records (median number of repeated measurements per woman: 10, interquartile range: 8, 11) and the corresponding gestational age and date [32]. GWG was defined as the difference between pre-pregnancy weight and the weight measured closest to the delivery date and included in the analyses as a continuous variable (kg).

### Dietary information

At 25 weeks of gestation, women in the DNBC were asked to report the frequency of intake of approximately 360 foods and beverages during the previous four weeks using a FFQ. Individual food items were quantified into grams per day based on assumptions of standard portion sizes [33], and related food items were aggregated into food groups. Quantification of nutrients relied on the use of food composition tables [34]. Food group and nutrient calculations were performed using the nutritional calculation software FoodCalc [35]. The FFQ contained detailed information on frequency of fish consumption (of more than 10 types of fish), either as a hot meal or as a cold meal (e.g. sandwiches). The dietary exposures for the present study were total fish, lean fish and fatty fish. Salmon, herring, mackerel, trout, and Greenland halibut were classified as fatty fish, while cod, pollack, plaice, flounder, garfish, and comparable species, as well as shellfish were classified as lean fish [36]. Consumption of fish as a hot meal and consumption of fish in a cold meal were combined using assumptions on standard portion sizes [37], and quantified in grams (g).

In the ALSPAC cohort, dietary information was obtained using FFQs at 32 weeks of gestation. Three questions were used to assess the participants’ intake of fish: “How many times nowadays do you eat (a) white fish (cod, haddock, plaice, fish fingers, etc.), (b) dark or oily fish (tuna, sardines, pilchards, mackerel, herring, kippers, trout, salmon, etc.), and (c) shellfish (prawns, crabs, cockles, mussels etc.)?”. Response was chosen from five predefined categories: Never or rarely; once in 2 weeks; 1–3 times per week; 4–7 times per week; and more than once a day. Using assumptions on standard portion sizes, intake of fish was quantified in grams. For the present study, lean fish was defined as the sum of white fish and shellfish, while fatty fish was defined as intake of dark or oily fish. In both cohorts we included intake of fish in the analyses as continuous variables (Number of [150 g] servings per week).

### Genetic information

The detailed procedure for genotyping in DNBC and ALSPAC has been described elsewhere [28;38]. For the current study, we included a total of 37 SNPs, of which 32 have been found consistently associated with a higher BMI in GWAS among non-pregnant women and men [39], and 5 variants associated to type 2 diabetes which have been suggested to play a role specifically in relation to GWG in a candidate gene study by Stuebe et al. (2010) [19] **(S1 Table)**.

For each subject, the 37 SNPs were coded 0, 1 or 2 according to number of BMI or GWG associated risk alleles. The 32 BMI associated SNPs were summed into a genetic risk score (GRS) with higher score indicating higher genetic predisposition to obesity.

### Covariates

From the interview in gestational week 16, we obtained information from the DNBC about the mother’s self-reported pre-pregnancy weight and height, maternal age, parity, social-occupational status (defined by education and occupation), physical activity, smoking and alcohol intake during pregnancy. Height and pre-pregnancy weight were used to calculate pre-pregnancy BMI (kg/m^2^), which together with age and total energy intake per day, were used as continuous variables. The following covariates were used as categorical variables: parity as 0, 1, or ≥ 2 children; social-occupational status as high-, intermediate- or low; physical activity as 0 min/week, 1–90 min/week or >90 min/week; smoking during pregnancy as non-smokers, ≤10 cigarettes/day, >10 cigarettes/day; alcohol intake as 0 units/week, ½-3 units/week or >3 units/week. Gestational age at birth was obtained from the Medical Birth Register and used as a continuous variable.

In ALSPAC, the mothers received four postal self-completion questionnaires during pregnancy [40]. Self-reported pre-pregnancy weight and height were used to calculate pre-pregnancy BMI, which together with maternal age and gestational age at birth (obtained from obstetrical records), were included in the analyses as continuous variables. The categorical covariates assessed during pregnancy, were: maternal smoking, alcohol consumption, physical activity, educational status, and occupational status (smoking: non-smokers, ≤ 15 cigarettes/day, >15 cigarettes/day; alcohol: 0 glass/week, 1–7 glasses/week, >7 glasses/week; physical activity: 0 hours/week, 1–3 hours/week, >3 hours/week, respectively; educational status: no qualifications, secondary certificate, ordinary-level qualification, advanced-level qualification, teaching or nursing qualification, university degree; occupational status: unskilled occupation, partly skilled occupation, manual skilled occupation, non-manual skilled occupation, managerial and technical occupation, professional occupations; parity (from obstetrical record): 0, 1, or ≥2 births).

### Statistical analysis

#### Exploratory analyses

The initial set of the analyses were performed in the DNBC sample. Linear regression analyses were used to investigate associations between dietary exposures (total, lean and fatty fish) and GWG, with adjustment for pre-pregnancy BMI, maternal age, gestational age at birth, parity, social-occupational status, physical activity, smoking and alcohol intake during pregnancy. Furthermore, we analysed the main effect of the GRS on GWG.

In further analyses, results were also adjusted for total energy intake, and these models must be interpreted differently. Inclusion of total energy introduces a substitution model where interpretation-wise, given a fixed total energy intake, a higher intake of fish must be accompanied by a lower intake of energy from other non-specified sources. In the analyses without total energy intake, fish consumption may in this sense vary without concomitant differences in the diet.

To examine interaction between genetic predisposition scores and fish intake in relation to GWG, we correspondingly added the GRS variable as well as the interaction terms (GRS × fish intake) to the model.

“Case status” (obese vs. non-obese/random sample) of the subjects was taken into account in an additional full analysis set in order to investigate any possible effect modification (two-way and three-way interactions, respectively).

Finally, we performed all analyses using the individual SNPs. As Stuebe et al. have suggested that the following T2D associated SNPs may play a role specifically in relation to GWG (*KCNQ1* rs2237892, *PPARG* rs1801282, *TCF2* rs4430796, *G6PC2* rs560887, *TSPAN8* rs7961581) [19], analyses of these SNPs were initially performed without adjustment for multiple testing, while Bonferroni adjustment was performed in analyses of the remaining 32 BMI associated SNPs.

P-values ≤ 0.05 were regarded as statistically significant. The Bonferroni correction was implemented by multiplying the unadjusted p-values with the number of SNPs tested, up to a maximum adjusted p-value of 1. If the adjusted p-value was still significant (P<0.05) the null hypothesis of no corresponding effect was rejected.

#### Replication analyses

In ALSPAC, the main effect of dietary fish (total, lean and fatty fish) on GWG was analyzed as described above. Moreover, we attempted replication of all analyses including genetic material (i.e. main effect and interaction analyses) if they were statistically significant in the DNBC.

#### Sensitivity analyses

Since women's pre-pregnancy BMI is a strong indicator of GWG, we included this variable in the primary model. However, it has been suggested that adjustment for baseline values in the analyses of changes can cause bias [41]. Thus, we also performed all analyses without adjustment for baseline BMI, to assess whether this caused any differences in the results.

Finally, to investigate whether any significant associations were mediated through growth rate of the child, supplementary analyses were conducted with adjustment for birth weight.

Analyses of DNBC data were performed using the statistical software package Stata 12, and analyses of ALSPAC data were performed using Stata 13 (StataCorp LP, College Station, Texas, USA; www.stata.com).

## Results

For the current study, we had information on genetic variants, dietary fish, anthropometry and covariates on a total of 1,128 non-obese and 990 obese individuals from the exploratory cohort (DNBC). In addition to this, we had information on 4,603 non-obese and 238 obese individuals from the replication cohort (ALSPAC). Information on intake of fish, GWG, GRS and covariates can be found in **Table 1.** No substantial difference in median GRS between the group of obese and the random sample in DNBC can be seen in Table 1. However, there was a statistically significant mean difference between the groups, and a statistically robust association between the GRS and pre-pregnancy BMI (β = 0.06, P = 3.5e-16). The DNBC participants had a lower median GWG than ALSPAC participants, as indicated in **Table 1** this difference can partly be explained by the larger proportion of obese participants in DNBC. Furthermore, DNBC had a lower intake of fish and a higher total energy intake than ALSPAC participants.

**Table 1:** Characteristics of all participants, and divided into obese and non-obese. Reported as median (5–95 percentiles) unless otherwise stated.

	Exploratory cohort (DNBC)			Replication cohort (ALSPAC)		
	All	Obese	Non-obese	All	Obese	Non-obese
**N**	2,118	990	1,128	4,841	238	4,603
**Maternal age at conception, years**	30 (24, 38)	30 (24, 38)	30 (24, 38)	28 (21, 36)	28 (20, 37)	28 (21, 36)
**BMI**	30.1 (19.4, 41.0)	35.7 (33.4, 43.0)	22.5 (18.7, 29.7)	22.2 (18.7, 29.8)	32.7 (30.2, 41.33)	22.0 (18.6, 27.4)
**GWG, kg**	13 (4, 25)	10 (3, 25)	15 (8, 25)	16.6 (8.2, 25.5)	13.1 (0.8, 24.3)	16.7 (8.7, 25.6)
**Gestational age at birth, weeks**	40 (37, 42)	40 (37, 42)	40 (37, 42)	40 (38, 42)	40 (37, 42)	40 (38,42)
**Primiparous, %**	49.8	49.2	50.4	50.6	42	51
**Total energy, kJ/day**	9,640 (6,068, 14,518)	9,174 (5,783, 14,304)	9,951 (6,360, 14,618)	7,157 (4,407, 10,579)	6,483 (3,886, 10,184)	7,181 (4,444, 10,622)
**Fatty fish, weekly servings**	0.0 (0.0, 1.1)	0.0 (0.0, 0.9)	0.0 (0.0, 1.3)	0.4(0, 1.4)	0.4 (0, 1.4)	0.4 (0, 1.4)
**Lean fish, weekly servings**	0.5 (0, 2.1)	0.3 (0, 2)	0.5 (0.0, 2.4)	0.7 (0, 2.0)	0.4 (0, 2.9)	0.7 (0, 2.0)
**Total fish, weekly servings**	0.6 (0, 3.0)	0.5 (0, 2.8)	0.8 (0.0, 3.4)	1.6 (0, 3.5)	1.1 (0, 4.4)	1.6 (0, 3.5)
**n-3 fatty acids (g/day)**	0.6 (0.3, 1.2)	0.6 (0.3, 1.1)	0.7 (0.4, 1.2)	NA	NA	NA
**n-6 fatty acids (g/day)**	2.5 (1.2, 4.3)	2.5 (1.3, 4.3)	2.6 (1.3, 4.3)	NA	NA	NA
**Non-smokers, %**	85.1	85.5	84.7	81.9	80.2	82
**Low social-occupational status, %**	12.4	16.3	8.9	16.4	22.7	16.1
**Alcohol, % non-drinkers**	60.3	69.2	52.2	43.4	50.8	43
**Physically inactive, %**	64.9	67.5	62.4	32.6	31.1	32.7
**GRS**	28 (23, 34)	29 (23, 34)	28 (22, 34)	NA	NA	NA

Abbreviations: BMI, body mass index; GWG, gestational weight gain; GRS, sum of the BMI-associated risk alleles

### Dietary fish and gestational weight gain

In analysis of all women in the DNBC sample, we found that each additional weekly portion of fatty fish was associated with a GWG of 0.58 kg (95% CI: 0.16, 0.99, P = 0.006). The same pattern was observed for both obese and non-obese women, but the association was not statistically significant in the latter group. In analyses of total dietary fish and lean fish, the direction of the association was consistent with fatty fish, but weaker and not statistically significant. Furthermore, we found no evidence for an interaction with obesity status, and further adjustment for total energy intake had very limited influence on the observed associations **(Table 2)**.

**Table 2:** Association between intake of fish (weekly servings) and subsequent gestational weight gain (kg).

	All			Obese			Non-obese			
	β	95% CI	P	β	95% CI	P	β	95% CI	P	P for
										Interaction^3^
***Exploratory cohort***	*** ***	*** ***								
***(DNBC)***										
**Basic adjustments**^1^										
*Fatty fish*	0.58	(0.16, 0.99)	<0.01	0.48	(-0.19, 1.15)	0.16	0.65	(0.13, 1.16)	0.01	0.10
*Lean fish*	0.16	(-0.11, 0.42)	0.25	0.15	(-0.27, 0.58)	0.48	0.16	(-0.16, 0.49)	0.33	0.85
*Total fish*	0.14	(-0.06, 0.34)	0.18	0.27	(-0.06, 0.61)	0.11	0.05	(-0.20, 0.30)	0.70	0.16
**Energy adjusted**^2^										
*Fatty fish*	0.47	(0.06, 0.89)	0.03	0.37	(-0.30, 1.04)	0.28	0.56	(0.04, 1.08)	0.03	0.10
*Lean fish*	0.06	(-0.21, 0.32)	0.68	0.04	(-0.39, 0.47)	0.85	0.08	(-0.25, 0.41)	0.65	0.85
*Total fish*	0.07	(-0.14, 0.27)	0.52	0.20	(-0.14, 0.54)	0.25	-0.02	(-0.27, 0.23)	0.90	0.16
***Replication cohort***	*** ***	*** ***								
***(ALSPAC)***										
**Basic adjustments**										
*Fatty fish*	0.14	(-0.09, 0.37)	0.24	0.02	(-1.47, 1.50)	0.98	0.17	(-0.062, 0.399)	0.15	0.56
*Lean fish*	-0.07	(-0.23, 0.10)	0.42	-1.05	(-1.98, -0.13)	0.03	0.02	(-0.145, 0.185)	0.81	<0.01
*Total fish*	0.00	(-0.12, 0.12)	0.99	-0.60	(-1.30, 0.11)	0.10	0.05	(-0.063, 0.167)	0.38	<0.01
**Energy adjusted**										
*Fatty fish*	0.05	(-0.18, 0.29)	0.66	-0.17	(-1.68, 1.344)	0.83	0.09	(-0.15, 0.32)	0.47	0.56
*Lean fish*	-0.16	(-0.32, 0.01)	0.07	-1.19	(-2.13, -0.26)	0.01	-0.06	(-0.23, 0.11)	0.46	<0.01
*Total fish*	-0.06	(-0.18, 0.06)	0.29	-0.73	(-1.45, -0.02)	0.04	-0.01	(-0.13, 0.11)	0.88	0.01

^1^Calculated using linear regression. Adjusted for pre-pregnancy BMI, maternal age at conception, gestational age at birth, parity, social-occupational status, physical activity, smoking and alcohol intake during pregnancy.

^2^Adjusted for the above covariates as well as total energy intake.

^3^ Obesity status interaction

In analyses of all women in ALSPAC, we found no evidence of an association between intake of fish and GWG. However, in analyses of fatty fish, we found the same direction in associations estimate as in the DNBC sample (0.14 kg [95% CI: -0.09, 0.37, P = 0.243] per additional portion). Furthermore, among obese participants, we found that consumption of lean fish was associated with a lower GWG (-1.05 kg [95% CI: -1.98, -0.13, P = 0.026] per additional portion). Finally, contrary to the DNBC analyses, we found a statistically robust obesity status interaction for lean and total fish in ALSPAC, indicating that consumption of fish was associated with lower GWG among obese, but higher GWG among non-obese. Additional adjustment for total energy intake also had a limited influence on the observed associations in ALSPAC **(Table 2)**.

### Genetic predisposition scores and gestational weight gain

We found no association between the GRS or selected T2D variants and GWG in the DNBC **(Table 3).** Likewise, after adjustment for multiple testing, we found no strong evidence that the 32 individual BMI associated SNPs were associated with GWG **(S2 Table).**

**Table 3:** Association between genetic risk score and selected SNPs in relation to gestational weight gain in the DNBC.

	All			Obese			Non-obese			
	β^3^	95% CI	P	β	95% CI	P	β	95% CI	P	P for
										Interaction^4^
GRS^1^	-0.01	(-0.07, 0.05)	0.73	-0.07	(-0.16, 0.03)	0.19	0.03	(-0.05, 0.11)	0.41	0.11
rs1801282i^2^	0.33	(-0.09, 0.75)	0.13	0.33	(-0.32, 0.98)	0.32	0.36	(-0.18, 0.90)	0.19	0.96
rs2237892	0.04	(-0.58, 0.67)	0.89	-0.51	(-1.47, 0.46)	0.30	0.53	(-0.28, 1.33)	0.20	0.12
rs4430796	0.00	(-0.30, 0.31)	0.99	-0.05	(-0.52, 0.43)	0.84	0.11	(-0.28, 0.49)	0.59	0.62
rs560887	-0.28	(-0.61, 0.05)	0.09	-0.51	(-1.03, 0.01)	0.06	-0.10	(-0.51, 0.31)	0.63	0.20
rs7961581i	0.21	(-0.15, 0.57)	0.25	0.66	(0.09, 1.22)	0.02	-0.12	(-0.57, 0.33)	0.60	0.05

^1^ Sum of BMI associated risk alleles

^2^An "i" following the rs-number indicates that imputed SNP information was used.

^3^Calculated using linear regression. Adjusted for pre-pregnancy BMI, maternal age at conception, gestational age at birth, parity, social-occupational status, physical activity, smoking and alcohol intake during pregnancy.

^4^ Obesity status interaction

### Interaction between genetic variants and dietary fish

Interaction estimates for GRS and the five selected T2D variants can be found in **Table 4**. We found no evidence of interaction between GRS and total fish or subgroups of fish in relation to GWG **(Table 4)**.

**Table 4:** Gene × fish interactions in relation to gestational weight gain, presented in kg per additional risk allele for each weekly serving of fish.

	All			Obese			Non-obese			
	β^3^	95% CI	P	β	95% CI	P	β	95% CI	P	P
										interaction^4^
***Exploratory cohort***	*** ***	*** ***								
***(DNBC)***										
**Fatty fish**										
GRS^1^	0.08	(-0.06, 0.21)	0.28	-0.06	(-0.29, 0.18)	0.64	0.13	(-0.03, 0.29)	0.12	0.23
rs1801282i^2^	-1.60	(-2.73, -0.47)	<0.01	-1.84	(-3.78, 0.11)	0.06	-1.67	(-3.03, -0.32)	0.02	0.99
rs2237892	0.86	(-0.33, 2.06)	0.16	1.18	(-1.29, 3.65)	0.35	0.62	(-0.68, 1.91)	0.35	0.67
rs4430796	0.59	(-0.11, 1.28)	0.10	0.53	(-0.86, 1.92)	0.45	0.69	(-0.08, 1.46)	0.08	0.86
rs560887	-0.13	(-0.92, 0.66)	0.74	-0.40	(-1.95, 1.16)	0.62	-0.19	(-1.06, 0.68)	0.67	0.89
rs7961581i	0.37	(-0.45, 1.18)	0.38	-0.74	(-2.49, 1.02)	0.41	0.90	(0.03, 1.78)	0.04	0.11
**Lean fish**										
GRS	0.04	(-0.05, 0.13)	0.37	-0.01	(-0.17, 0.16)	0.94	0.05	(-0.05, 0.15)	0.30	0.52
rs1801282i	-1.11	(-1.70, -0.53)	<0.01	-0.54	(-1.60, 0.52)	0.32	-1.57	(-2.25, -0.89)	<0.01	0.26
rs2237892	0.29	(-0.67, 1.25)	0.55	1.43	(-0.38, 3.23)	0.12	-0.48	(-1.57, 0.61)	0.39	0.08
rs4430796	0.19	(-0.22, 0.60)	0.36	-0.41	(-1.10, 0.29)	0.25	0.60	(0.11, 1.08)	0.02	0.42
rs560887	-0.04	(-0.53, 0.44)	0.86	-0.12	(-0.10, 0.76)	0.79	-0.08	(-0.65, 0.48)	0.77	0.35
rs7961581i	0.07	(-0.44, 0.58)	0.79	-0.31	(-1.14, 0.51)	0.46	0.31	(-0.31, 0.94)	0.33	0.41
**Total fish**										
GRS	0.01	(-0.05, 0.08)	0.65	-0.02	(-0.14, 0.10)	0.732	0.02	(-0.06, 0.09)	0.65	0.60
rs1801282i	-0.83	(-1.29, -0.37)	<0.01	-0.62	(-1.41, 0.18)	0.129	-1.10	(-1.65, -0.55)	<0.01	0.08
rs2237892	0.19	(-0.43, 0.80)	0.55	0.95	(-0.32, 2.21)	0.143	-0.27	(-0.95, 0.40)	0.43	0.06
rs4430796	0.02	(-0.30, 0.34)	0.90	-0.16	(-0.70, 0.38)	0.559	0.13	(-0.27, 0.52)	0.53	0.02
rs560887	0.15	(-0.22, 0.51)	0.43	-0.17	(-0.83, 0.50)	0.617	0.24	(-0.18, 0.66)	0.26	0.98
rs7961581i	-0.06	(-0.44, 0.32)	0.75	-0.29	(-0.96, 0.37)	0.386	0.08	(-0.37, 0.52)	0.74	0.25
***Replication cohort***	*** ***	*** ***								
***(ALSPAC)***										
**Fatty fish**										
rs1801282i	-0.05	(-0.51, 0.41)	0.83	0.27	(-2.69, 3.22)	0.86	-0.02	(-0.48, 0.43)	0.92	0.58
**Lean fish**										
rs1801282i	-0.17	(-0.52, 0.19)	0.36	-2.10	(-4.41, 0.21)	0.07	-0.11	(-0.46, 0.24)	0.55	0.04
**Total fish**										
rs1801282i	-0.09	(-0.33, 0.15)	0.48	-0.85	(-2.50, 0.79)	0.31	-0.05	(-0.29, 0.18)	0.65	0.12

^1^ Sum of BMI associated risk alleles

^2^ An "i" following the rs-number indicates that imputed SNP information was used.

^3^SNP (or GRS) × dietary fish product terms were calculated using linear regression. Adjusted for pre-pregnancy BMI, maternal age at conception, gestational age at birth, parity, social-occupational status, physical activity, smoking and alcohol intake during pregnancy.

^4^ Obesity status interaction

**Fig 1:**
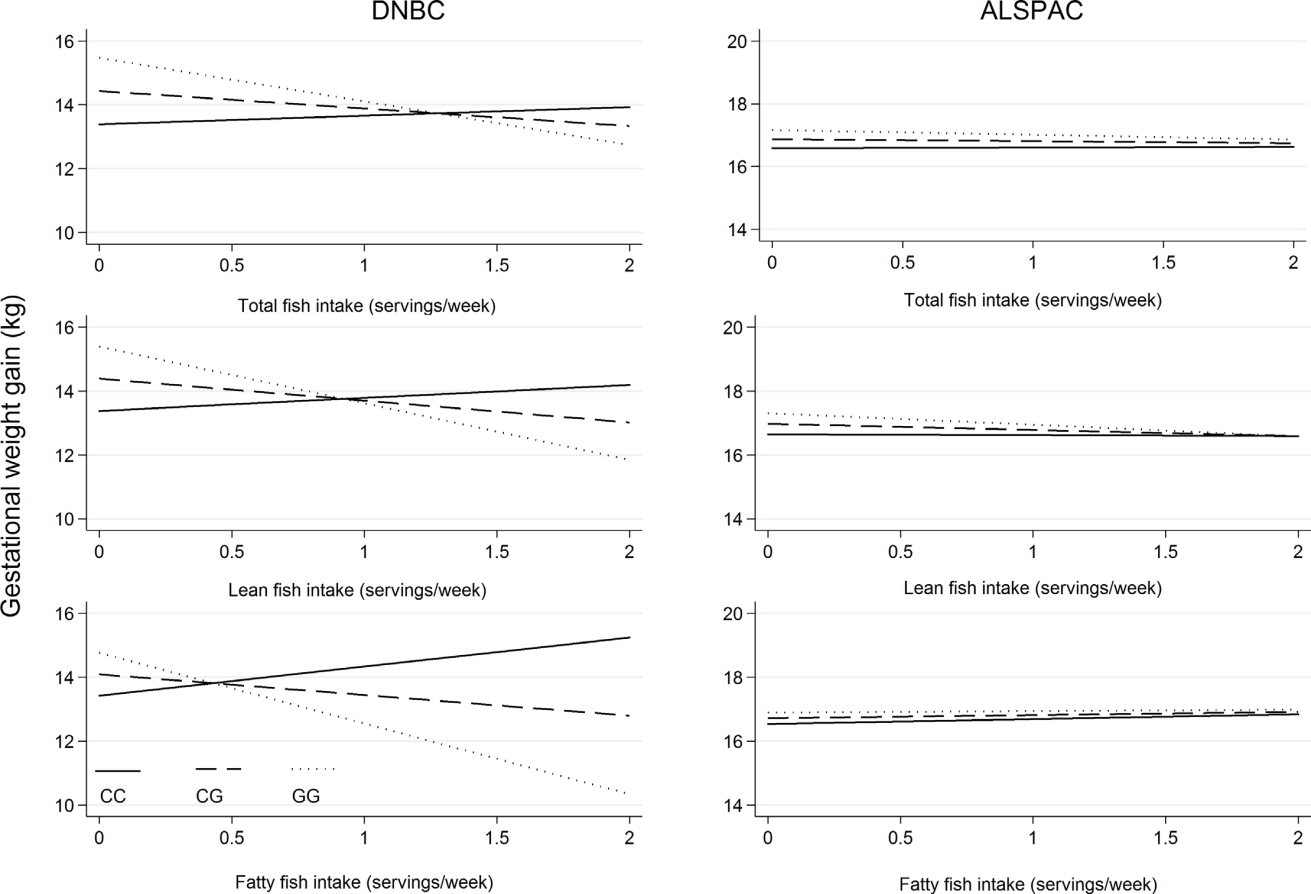
Interaction between PPARG rs1801282 and dietary fish in relation to gestational weight gain in the DNBC and ALSPAC. Adjusted for pre-pregnancy BMI, maternal age at conception, gestational age at birth, parity, social-occupational status, physical activity, smoking and alcohol intake during pregnancy.

When studying the five T2D variants, we found that each additional G-allele of the *PPARG* rs1801282, was associated with a GWG of -0.83 kg (95% CI: -1.29, -0.37, P = 0.0004) per weekly portion of total dietary fish, -1.60 kg (95% CI: -2.73, -0.47, P = 0.006) per weekly portion of fatty fish, and -1.11 kg (95% CI: -1.70, -0.53, P = 0.0002) per weekly portion of lean fish. In an analysis adjusting for multiple testing, this interaction remained statistically significant for total dietary fish (P_Bonferroni_ = 0.015) and lean fish (P_Bonferroni_ = 0.007), but not for fatty fish (P_Bonferroni_ = 0.211). We were unable to replicate this interaction in the ALSPAC cohort. However, the direction of the estimates was consistent with DNBC both for fatty, lean and total fish (**Table 4**). A graphic illustration of this interaction can be seen in **Fig 1**. In the DBNC the figure shows a tendency to lower GWG with higher fish intake among GG and GC allele carriers, while the opposite is seen for CC carriers. The same pattern can be seen for all types of fish, but strongest for fatty fish. In ALSPAC, no clear differences between *PPARG* rs1801282 genotypes were seen in the relationship between dietary fish and GWG.

*KCNQ1* rs2237892, *TCF2* rs4430796, *G6PC2* rs560887 and *TSPAN8* rs7961581 showed no statistically significant interaction with dietary intake of fish in DNBC **(Table 4**). A complete overview of results related to interactions between specific SNPs and dietary fish intake in the DNBC sample can be found in **S3–S5 Tables.**

### Sensitivity analyses

We also performed analyses without adjustment for baseline BMI and with adjustment for birth weight, but this had no substantial influence on the observed associations (results not shown).

## Discussion

In the explorative analyses conducted in the DNBC sample, we found that dietary intake of fatty fish was associated with a higher GWG. In analyses of total fish and lean fish, the direction of the association was consistent with fatty fish, but not statistically significant. However, in analyses of all women in ALSPAC, we were not able to replicate an association between intake of fish and GWG. In the DNBC, we found no evidence of association between the GRS and GWG and no statistically significant interactions between the GRS and dietary fish intake. An interaction between the *PPARG* rs1801282 variants and dietary fish was observed, indicating that dietary fish was associated with a lower GWG among GG and GC carriers, while the opposite was seen for CC genotype carriers. However, we were unable to replicate this finding in the ALSPAC cohort.

We found no other studies investigating the association between intake of fish and GWG. Results from a randomized controlled study among men and non-pregnant women suggest that increasing fish intake in an energy-restrictive diet can promote weight loss [8], while prospective cohort studies have shown that fish consumption has no appreciable association with body-weight gain (31).

Contrary to this, the results from the DNBC cohort suggest that a higher intake of fatty fish may be associated with higher GWG, and though not statistically significant, the same direction in the estimate was seen in ALSPAC. In line with these results, previous studies have suggested that maternal fish intake is associated with a lower frequency of low birth weight [4;42]. This could be due to a greater intake of n-3 fatty acids, but also the fact that fish contains protein of high biological value that could play a central role in fetal growth [16;43]. Thus, our results could be explained by a greater fetal growth rate among women with the highest fish intake. However, further adjustment for birth weight in sensitivity analyses did not change the results in this study, suggesting that our results were driven by maternal weight gain.

We found no prior studies that examined the relationship between genetic predisposition to obesity (measured using GRS) in relation to GWG with the selection of SNPs included in our study. However, Lawlor et al. (2011) examined the association between a GRS of four BMI variants (*FTO* rs9939609, *MC4R* rs17782313, *TMEM18* rs6548238, and *GNPDA2* rs10938397) and GWG, and found no association when observing the entire pregnancy period [29]. Furthermore, we included 5 variants associated with T2D which have been suggested to play a role specifically in relation to GWG in an explorative candidate gene study by Stuebe et al. (2010) [19]. We were unable to replicate any of these findings **(Table 3),** and as Stuebe et al. performed an exploratory candidate gene study (including 27 variants) without adjustment for multiple testing this could indicate false positive results. Nevertheless, in this context it should be mentioned that we conducted analyses using an additive genetic model while Stuebe et al. used a general model (allowing non-additive associations) and thus, the results from the two studies cannot be directly compared.

In the DNBC sample, we found preliminary evidence of interaction between the *PPARG* rs1801282 and dietary fish, indicating that the G-allele of the rs1801282 variant attenuates the association between dietary fish and GWG. We could not replicate this finding in the ALSPAC cohort, although the direction of the estimates was the same. We found no previous studies examining interaction between *PPARG* rs1801282 and dietary fish, but studies have previously shown that this variant interacts with fat intake in relation to obesity among non-pregnant woman and men [44–46]. Thus, it is possible that the *PPARG* rs1801282 dietary fish interaction observed in the DNBC is due to the fact that women with a high consumption of fish may have a different fat intake, both in terms of total fat intake and specific fatty acids. However, as we were not able to replicate this finding it could very well be a false positive result.

The strengths of our study include the use of data from a large nested case-cohort study with information on dietary intake from validated FFQs [47–49], GWG, as well as information on several potential confounders. We had information on 32 SNPs found to be consistently associated with BMI allowing us to calculate a GRS. The genetic information along with the case-cohort design made it possible for us to investigate interaction between dietary fish and genetic predisposition to obesity among both obese and non-obese women.

Our study also has some limitations. As most other methods of quantifying dietary intake, FFQs are subject to inaccuracies particularly as a tool to measure the total energy content of the diet [50;51]. Hence, measurement error related to dietary intake could have biased the results towards the null and led to wider confidence intervals. Moreover, the participants from the DNBC had a low fish consumption with a median intake of 0.6 portions a week (5–95% percentile: 0.0 to 3.0), and the limited findings could be due to a low variation in exposure.

Adjustment for total energy intake excludes the possibility to investigate satiety effects of fish intake, and therefore we did not include total energy intake in the primary model. However, differences in total energy intake can result in extraneous variation. Thus, failure to adjust for total energy intake can lead to misclassification of the biologically important variation in fish consumption as the same amount of fish intake should be expected to have a larger effect on obesity in a small person than in a large person. Also, adjustment for total energy intake may not only reduce extraneous variation, but also improve the validity of estimated fish intake by in part adjusting for measurement error. For these reasons, we adjusted for total energy intake in further analyses, but doing so did not substantially change the results.

In the DNBC, we relied on self-reported information on GWG and pre-pregnancy BMI. It has been shown that self-reported GWG is likely to produce association estimates similar to those based on GWG data from birth certificates [52]. However, misreporting could potentially bias our results in either direction if reporting of GWG is related to intake of fish, but differential misclassification is unlikely. Furthermore, as in other epidemiological studies, we cannot rule out that unmeasured or residual confounding have affected our results. In this regard, we did not explore confounding or substitution with specific nutrient or food items, and consequently we cannot rule out that this could affect our results.

Some limitations also apply specifically for analyses including GRS. The score of BMI associated risk alleles was based on established BMI associated variants. Nevertheless, these variants only explain a very limited proportion of the total variation in obesity (<2%). Furthermore, the SNPs included in this paper were identified through review of GWAS published until 2010. Since then, several additional variants have been identified [18]. However, the contribution of these variants to the total genetic variation in obesity is marginal. The use of a GRS based on genetic variants associated with current status of BMI in cross-sectional GWAS may not be applicable when analysing GWG. In this regard, we have previously shown that although this GRS is strongly associated with a higher weight status among non-pregnant individuals in cross-sectional data, it is not associated with weight gain [53], and the same was seen in relation to GWG in the present study (**Table 3**). In addition, the estimates from the GRS × dietary fish interaction analyses can be described as the average interaction effect per BMI associated risk allele. Thus, for the method to be able to capture interaction effects of multiple SNPs there needs to be a general consistency between the direction of the adiposity-related main effect of the included SNPs and the direction of the SNP × dietary fish interaction effect in relation to GWG. This assumption is not necessarily correct, perhaps explaining the lack of interactions observed in our study.

We used ALSPAC as a replication cohort, but this cohort is different from the DNBC sample in several ways. First, dietary information was collected in different periods of the pregnancy in the two cohorts. In DNBC, FFQ information was collected in gestational week 25, whereas the same information was collected at week 32 in the ALSPAC cohort. Second, different classifications of dietary fish were used in the FFQs. While DNBC had information on more than 10 different types of fish, divided according to warm and cold meals, ALSPAC had a total of three questions related to fish consumption. Third, even though we tried to mimic the design of DNBC in the ALSPAC cohort, the number of obese participants in the DNBC sample is much higher due to the nested case-cohort design. All these factors could potentially be a contributing factor to the limited replication success. Finally, our analyses were conducted in a population of North European descent, and results may not be generalizable to other ethnic groups.

In conclusion, we found no consistent evidence for an association between fish consumption and GWG. Furthermore, the results indicate that GWG has little or no dependency on overall molecular genetic predisposition to obesity, and we found no replicable interaction between fish consumption and individual genetic variants. However, some indication of interaction between *PPARG* rs1801282 and dietary fish was observed, but further replication of this finding in an even larger setting is needed.

## Supporting Information

S1 Fig Flowchart showing the selection of participants from the DNBC. (DOCX)

S2 Fig Flowchart showing the selection of participants from the ALSPAC. (DOCX)

S1 Table Information on the 37 SNPs included in this study. (DOCX)

S2 Table Associations between SNPs and gestational weight gain. (DOCX)

S3 Table SNP × total dietary fish interaction in relation to gestational weight gain. (DOCX)

S4 Table SNP × fatty fish interaction in relation to gestational weight gain. (DOCX)

S5 Table SNP × lean fish interaction in relation to gestational weight gain. (DOCX)

## Abbreviations

ALSPAC: Avon Longitudinal Study of Parents and Children
CI: confidence interval
DNBC: Danish National Birth Cohort
FFQ: food frequency questionnaire
GWG: gestational weight gain
GWAS: genome-wide association studies
SNP: single-nucleotide polymorphism.
