# Supplementary material for: Association between Maternal Fish Consumption and Gestational Weight Gain: Influence of Molecular Genetic Predisposition to Obesity
Source: PLoS One. 2016 Mar 1;11(3):e0150105. doi: 10.1371/journal.pone.0150105 (PMC4773113; doi:10.1371/journal.pone.0150105)
Supplement: S1 Fig — (DOCX) [file pone.0150105.s001.docx]

**S1 Fig. Flowchart showing the selection of participants from the DNBC.**

92,274 women with 100,418 pregnancies were enrolled in the cohort (1996 to 2003)

**Control group**

**n = 1,128** normal weight and obese women

**Obese group**

**n = 990** severely obese women

**Women eligible for case-cohort sampling**:

- - - Blood sample taken during pregnancy
    - Successful buffy coat extraction
    - Live born singleton
    - Participated in interview 1
    - Information on pre-pregnancy BMI available

**n = 67,853**

**Cohort group**

Randomly selected women from the reaming cohort

**n = 2,450**

**Obese group**

The 3.6 % with the largest residuals from the regression of BMI on age and parity

**n = 2,449**

**Obese and control group women with information on**

- - - - Gestational weight gain
      - Information on genetic data
      - Information on diet and covariates
      - With a total energy intake between 2500 and 25000kj
