# Supplementary material for: Association between Maternal Fish Consumption and Gestational Weight Gain: Influence of Molecular Genetic Predisposition to Obesity
Source: PLoS One. 2016 Mar 1;11(3):e0150105. doi: 10.1371/journal.pone.0150105 (PMC4773113; doi:10.1371/journal.pone.0150105)
Supplement: S2 Fig — (DOCX) [file pone.0150105.s002.docx]

**S2 Fig. Flowchart showing the selection of participants from the ALSPAC.**

14,541 recruited participants, with expected date of delivery between 1 April 1991 and 31 December 1992

4,841 women with information on:

- - - - Gestational weight gain
      - Information on genetic data
      - Information on diet
      - Information on covariates
      - With a total energy intake between 2500 and 25000kj

**Non-obese group**

**BMI <30**

**n = 4,603**

**Obese group**

**BMI** ≥ **30**

**n = 238**
