# Supplementary material for: Association between Maternal Fish Consumption and Gestational Weight Gain: Influence of Molecular Genetic Predisposition to Obesity
Source: PLoS One. 2016 Mar 1;11(3):e0150105. doi: 10.1371/journal.pone.0150105 (PMC4773113; doi:10.1371/journal.pone.0150105)
Supplement: S1 Table — (DOCX) [file pone.0150105.s003.docx]

| **S1 Table. Information on the 37 SNP´s included in this study. The individual SNPs are sorted by refSNP (rs) number and grouped according to their associated trait** | | | | |
| --- | --- | --- | --- | --- |
| Trait | SNP | Nearest gene | Risk allele | Other allele |
| BMI | rs10146997 | NRXN3 | G | A |
| BMI | rs10508503 | PTER | C | T |
| BMI | rs10838738 | MTCH2 | G | A |
| BMI | rs10938397i | GNPDA2 | G | A |
| BMI | rs10968576 | LRRN6C | G | A |
| BMI | rs1121980i^1^ | FTO | A | G |
| BMI | rs11847697i | PRKD1 | T | C |
| BMI | rs12444979i | GPRC5B | C | T |
| BMI | rs13107325 | SLC39A8 | T | C |
| BMI | rs1424233 | MAF | A | G |
| BMI | rs1514175 | TNNI3K | T | C |
| BMI | rs1555543i | PTBP2 | C | A |
| BMI | rs17782313i | MC4R | C | T |
| BMI | rs1805081 | NPC1 | A | G |
| BMI | rs206936i | NUDT3 | G | A |
| BMI | rs2112347i | FLJ35779 | T | G |
| BMI | rs2241423 | MAP2K5 | G | A |
| BMI | rs2287019 | QPCTL | C | T |
| BMI | rs2568958 | NEGR1 | A | G |
| BMI | rs2890652i | LRP1B | C | T |
| BMI | rs29941 | KCTD15 | G | A |
| BMI | rs3810291i | TMEM160 | A | G |
| BMI | rs4712652 | LINC00340 | A | G |
| BMI | rs4771122i | MTIF3 | G | A |
| BMI | rs4929949i | RPL27A | C | T |
| BMI | rs543874i | SEC16B | G | A |
| BMI | rs6013029i | CTNNBL1 | T | G |
| BMI | rs6232 | PCSK1 | G | A |
| BMI | rs6602024i | PFKP | A | G |
| BMI | rs713586i | RBJ | C | T |
| BMI | rs7647305 | SFRS10 | C | T |
| BMI | rs9939609i | FTO | A | T |
| T2D (GWG) | rs1801282i | PPARG | G | C |
| T2D (GWG) | rs2237892 | KCNQ1 | C | T |
| T2D (GWG) | rs4430796 | TCF2 | A | G |
| T2D (GWG) | rs560887 | G6PC2 | A | G |
| T2D (GWG) | rs7961581i | TSPAN8 | T | C |
| *^1^ An "i" following the rs-number indicates that imputed SNP information was used* | | | | |
