# Supplementary material for: Association between Maternal Fish Consumption and Gestational Weight Gain: Influence of Molecular Genetic Predisposition to Obesity
Source: PLoS One. 2016 Mar 1;11(3):e0150105. doi: 10.1371/journal.pone.0150105 (PMC4773113; doi:10.1371/journal.pone.0150105)
Supplement: S2 Table — (DOCX) [file pone.0150105.s004.docx]

| **S2 Table. Associations between SNPs and gestational weight gain, presented in kg per additional minor allele** | | | | | | | | | | | | | | | |
| --- | --- | --- | --- | --- | --- | --- | --- | --- | --- | --- | --- | --- | --- | --- | --- |
|  | All | | | | | Obese | | | | | Non-obese | | | | |
| SNP | N | β^2^ | SE | P | P_B_^3^ | N | β | SE | P | P_B_ | N | β | SE | P | P_B_ |
| rs10146997 | 2,773 | -0.231 | 0.183 | 0.208 | 1 | 1,318 | 0.120 | 0.284 | 0.672 | 1 | 1,455 | -0.539 | 0.233 | 0.021 | 0.776 |
| rs10508503 | 2,772 | 0.257 | 0.276 | 0.351 | 1 | 1,317 | 0.748 | 0.446 | 0.093 | 1 | 1,455 | -0.060 | 0.339 | 0.86 | 1 |
| rs10838738 | 2,774 | -0.103 | 0.158 | 0.514 | 1 | 1,319 | 0.039 | 0.243 | 0.872 | 1 | 1,455 | -0.214 | 0.204 | 0.294 | 1 |
| rs10938397i^1^ | 2,776 | 0.169 | 0.157 | 0.282 | 1 | 1,319 | 0.110 | 0.243 | 0.651 | 1 | 1,457 | 0.136 | 0.201 | 0.498 | 1 |
| rs10968576 | 2,775 | 0.177 | 0.161 | 0.270 | 1 | 1,319 | 0.179 | 0.249 | 0.473 | 1 | 1,456 | 0.170 | 0.205 | 0.409 | 1 |
| rs1121980i | 2,776 | 0.007 | 0.154 | 0.965 | 1 | 1,319 | 0.033 | 0.238 | 0.889 | 1 | 1,457 | -0.003 | 0.197 | 0.989 | 1 |
| rs11847697i | 2,776 | 0.213 | 0.403 | 0.597 | 1 | 1,319 | 0.602 | 0.606 | 0.320 | 1 | 1,457 | -0.182 | 0.530 | 0.731 | 1 |
| rs12444979i | 2,776 | 0.165 | 0.236 | 0.484 | 1 | 1,319 | -0.256 | 0.373 | 0.491 | 1 | 1,457 | 0.542 | 0.296 | 0.067 | 1 |
| rs13107325 | 2,775 | 0.111 | 0.374 | 0.767 | 1 | 1,319 | -0.847 | 0.57 | 0.137 | 1 | 1,456 | 0.985 | 0.484 | 0.042 | 1 |
| rs1424233 | 2,772 | -0.144 | 0.153 | 0.346 | 1 | 1,317 | -0.085 | 0.24 | 0.724 | 1 | 1,455 | -0.201 | 0.194 | 0.301 | 1 |
| rs1514175 | 2,761 | -0.038 | 0.154 | 0.807 | 1 | 1,313 | -0.135 | 0.239 | 0.572 | 1 | 1,448 | 0.079 | 0.196 | 0.686 | 1 |
| rs1555543i | 2,776 | 0.267 | 0.155 | 0.085 | 1 | 1,319 | 0.302 | 0.24 | 0.209 | 1 | 1,457 | 0.217 | 0.198 | 0.273 | 1 |
| rs17782313i | 2,776 | -0.367 | 0.171 | 0.032 | 1 | 1,319 | -0.477 | 0.256 | 0.063 | 1 | 1,457 | -0.242 | 0.227 | 0.287 | 1 |
| rs1801282i | 2,776 | 0.326 | 0.214 | 0.128 | 1 | 1,319 | 0.328 | 0.332 | 0.323 | 1 | 1,457 | 0.364 | 0.275 | 0.186 | 1 |
| rs1805081 | 2,774 | -0.049 | 0.155 | 0.751 | 1 | 1,319 | -0.095 | 0.244 | 0.697 | 1 | 1,455 | -0.058 | 0.195 | 0.767 | 1 |
| rs206936i | 2,776 | -0.264 | 0.192 | 0.169 | 1 | 1,319 | -0.504 | 0.297 | 0.090 | 1 | 1,457 | -0.084 | 0.245 | 0.732 | 1 |
| rs2112347i | 2,776 | -0.110 | 0.162 | 0.498 | 1 | 1,319 | -0.398 | 0.253 | 0.116 | 1 | 1,457 | 0.127 | 0.206 | 0.538 | 1 |
| rs2237892 | 2,775 | 0.044 | 0.320 | 0.891 | 1 | 1,319 | -0.505 | 0.492 | 0.304 | 1 | 1,456 | 0.527 | 0.411 | 0.201 | 1 |
| rs2241423 | 2,774 | -0.181 | 0.180 | 0.316 | 1 | 1,319 | -0.255 | 0.284 | 0.369 | 1 | 1,455 | -0.099 | 0.227 | 0.663 | 1 |
| rs2287019 | 2,775 | -0.021 | 0.189 | 0.910 | 1 | 1,319 | -0.068 | 0.301 | 0.823 | 1 | 1,456 | -0.025 | 0.237 | 0.916 | 1 |
| rs2568958 | 2,772 | 0.099 | 0.156 | 0.524 | 1 | 1,316 | 0.117 | 0.242 | 0.63 | 1 | 1,456 | 0.166 | 0.199 | 0.403 | 1 |
| rs2890652i | 2,776 | 0.041 | 0.189 | 0.830 | 1 | 1,319 | -0.109 | 0.291 | 0.708 | 1 | 1,457 | 0.106 | 0.243 | 0.662 | 1 |
| rs29941 | 2,773 | 0.038 | 0.165 | 0.818 | 1 | 1,319 | 0.003 | 0.263 | 0.99 | 1 | 1,454 | 0.127 | 0.205 | 0.537 | 1 |
| rs3810291i | 2,776 | 0.097 | 0.183 | 0.595 | 1 | 1,319 | -0.074 | 0.284 | 0.796 | 1 | 1,457 | 0.226 | 0.234 | 0.335 | 1 |
| rs4430796 | 2,762 | 0.002 | 0.155 | 0.991 | 1 | 1,316 | -0.048 | 0.242 | 0.842 | 1 | 1,446 | 0.106 | 0.196 | 0.586 | 1 |
| rs4712652 | 2,772 | -0.120 | 0.155 | 0.439 | 1 | 1,317 | -0.144 | 0.241 | 0.549 | 1 | 1,455 | -0.035 | 0.198 | 0.858 | 1 |
| rs4771122i | 2,776 | 0.201 | 0.181 | 0.267 | 1 | 1,319 | -0.301 | 0.283 | 0.287 | 1 | 1,457 | 0.641 | 0.230 | 0.005 | 0.194 |
| rs4929949i | 2,776 | 0.078 | 0.158 | 0.620 | 1 | 1,319 | 0.069 | 0.244 | 0.777 | 1 | 1,457 | 0.001 | 0.203 | 0.995 | 1 |
| rs543874i | 2,776 | 0.043 | 0.180 | 0.813 | 1 | 1,319 | -0.142 | 0.271 | 0.600 | 1 | 1,457 | 0.213 | 0.238 | 0.369 | 1 |
| rs560887 | 2,774 | -0.281 | 0.167 | 0.093 | 1 | 1,318 | -0.512 | 0.267 | 0.055 | 1 | 1,456 | -0.100 | 0.207 | 0.630 | 1 |
| rs6013029i | 2,776 | 0.221 | 0.386 | 0.567 | 1 | 1,319 | 0.386 | 0.631 | 0.541 | 1 | 1,457 | 0.088 | 0.473 | 0.853 | 1 |
| rs6232 | 2,772 | 0.021 | 0.313 | 0.946 | 1 | 1,318 | 0.405 | 0.478 | 0.397 | 1 | 1,454 | -0.469 | 0.404 | 0.246 | 1 |
| rs6602024i | 2,776 | -0.189 | 0.240 | 0.432 | 1 | 1,319 | -0.520 | 0.364 | 0.152 | 1 | 1,457 | 0.163 | 0.313 | 0.603 | 1 |
| rs713586i | 2,776 | -0.245 | 0.152 | 0.106 | 1 | 1,319 | -0.049 | 0.238 | 0.838 | 1 | 1,457 | -0.395 | 0.192 | 0.039 | 1 |
| rs7647305 | 2,775 | 0.104 | 0.201 | 0.605 | 1 | 1,319 | -0.196 | 0.311 | 0.528 | 1 | 1,456 | 0.381 | 0.258 | 0.139 | 1 |
| rs7961581i | 2,776 | 0.210 | 0.182 | 0.249 | 1 | 1,319 | 0.655 | 0.286 | 0.022 | 0.819 | 1,457 | -0.120 | 0.228 | 0.600 | 1 |
| rs9939609i | 2,776 | 0.028 | 0.155 | 0.857 | 1 | 1,319 | 0.007 | 0.239 | 0.976 | 1 | 1,457 | 0.046 | 0.198 | 0.816 | 1 |
| *^1^An "i" following the rs-number indicates that imputed SNP information was used.*  *^2^Calculated using linear regression. Adjusted for pre-pregnancy BMI, maternal age at conception, gestational age at birth, parity, social-occupational status, physical activity, smoking and alcohol intake during pregnancy.*  *^3^Bonferroni adjusted P-value* | | | | | | | | | | | | | | | |
