# Supplementary material for: Association between Maternal Fish Consumption and Gestational Weight Gain: Influence of Molecular Genetic Predisposition to Obesity
Source: PLoS One. 2016 Mar 1;11(3):e0150105. doi: 10.1371/journal.pone.0150105 (PMC4773113; doi:10.1371/journal.pone.0150105)
Supplement: S3 Table — (DOCX) [file pone.0150105.s005.docx]

| **S3 Table. SNP × total dietary fish interaction in relation to gestational weight gain, presented in kg per additional risk allele for each serving of fish** | | | | | | | | | | | | | | | |
| --- | --- | --- | --- | --- | --- | --- | --- | --- | --- | --- | --- | --- | --- | --- | --- |
|  | All | | | | | Obese | | | | | Non-obese | | | | |
| SNP | N | β | SE | P | P_B_^1^ | N | β | SE | P | P_B_ | N | β | SE | P | P_B_ |
| rs10146997 | 2,095 | -0.175 | 0.174 | 0.314 | 1 | 975 | -0.714 | 0.317 | 0.024 | 0.899 | 1,120 | 0.134 | 0.198 | 0.497 | 1 |
| rs10508503 | 2,094 | 0.439 | 0.227 | 0.053 | 1 | 974 | 0.560 | 0.469 | 0.232 | 1 | 1,120 | 0.324 | 0.253 | 0.200 | 1 |
| rs10838738 | 2,096 | -0.026 | 0.168 | 0.877 | 1 | 976 | 0.119 | 0.300 | 0.692 | 1 | 1,120 | -0.084 | 0.195 | 0.665 | 1 |
| rs10938397i | 2,098 | 0.251 | 0.170 | 0.140 | 1 | 976 | 0.503 | 0.295 | 0.088 | 1 | 1,122 | 0.102 | 0.202 | 0.613 | 1 |
| rs10968576 | 2,097 | -0.070 | 0.165 | 0.668 | 1 | 976 | -0.311 | 0.279 | 0.266 | 1 | 1,121 | 0.074 | 0.198 | 0.709 | 1 |
| rs1121980i | 2,098 | -0.008 | 0.175 | 0.962 | 1 | 976 | -0.231 | 0.314 | 0.461 | 1 | 1,122 | 0.047 | 0.207 | 0.818 | 1 |
| rs11847697i | 2,098 | -0.136 | 0.454 | 0.764 | 1 | 976 | -0.379 | 0.793 | 0.632 | 1 | 1,122 | -0.034 | 0.536 | 0.950 | 1 |
| rs12444979i | 2,098 | -0.239 | 0.228 | 0.295 | 1 | 976 | -0.465 | 0.415 | 0.263 | 1 | 1,122 | -0.199 | 0.265 | 0.453 | 1 |
| rs13107325 | 2,097 | 0.201 | 0.379 | 0.596 | 1 | 976 | 0.353 | 0.709 | 0.618 | 1 | 1,121 | -0.075 | 0.437 | 0.864 | 1 |
| rs1424233 | 2,095 | -0.144 | 0.144 | 0.319 | 1 | 975 | -0.067 | 0.239 | 0.779 | 1 | 1,120 | -0.172 | 0.175 | 0.328 | 1 |
| rs1514175 | 2,086 | 0.024 | 0.153 | 0.874 | 1 | 972 | -0.090 | 0.259 | 0.73 | 1 | 1,114 | 0.077 | 0.188 | 0.681 | 1 |
| rs1555543i | 2,098 | 0.033 | 0.176 | 0.850 | 1 | 976 | 0.005 | 0.299 | 0.986 | 1 | 1,122 | 0.087 | 0.212 | 0.682 | 1 |
| rs17782313i | 2,098 | -0.080 | 0.165 | 0.626 | 1 | 976 | -0.348 | 0.311 | 0.262 | 1 | 1,122 | 0.047 | 0.190 | 0.806 | 1 |
| rs1801282i | 2,098 | -0.832 | 0.235 | 0.0004 | 0.015 | 976 | -0.617 | 0.406 | 0.129 | 1 | 1,122 | -1.101 | 0.279 | 0.0001 | 0.003 |
| rs1805081 | 2,096 | 0.208 | 0.167 | 0.212 | 1 | 976 | 0.185 | 0.277 | 0.504 | 1 | 1,120 | 0.117 | 0.205 | 0.567 | 1 |
| rs206936i | 2,098 | -0.084 | 0.222 | 0.706 | 1 | 976 | -0.197 | 0.392 | 0.616 | 1 | 1,122 | 0.065 | 0.261 | 0.805 | 1 |
| rs2112347i | 2,098 | -0.122 | 0.159 | 0.442 | 1 | 976 | -0.144 | 0.249 | 0.564 | 1 | 1,122 | -0.151 | 0.208 | 0.469 | 1 |
| rs2237892 | 2,097 | 0.187 | 0.312 | 0.549 | 1 | 976 | 0.947 | 0.646 | 0.143 | 1 | 1,121 | -0.273 | 0.344 | 0.428 | 1 |
| rs2241423 | 2,096 | 0.364 | 0.195 | 0.062 | 1 | 976 | 0.751 | 0.342 | 0.028 | 1 | 1,120 | 0.130 | 0.23 | 0.573 | 1 |
| rs2287019 | 2,097 | -0.275 | 0.203 | 0.176 | 1 | 976 | 0.009 | 0.345 | 0.979 | 1 | 1,121 | -0.436 | 0.243 | 0.072 | 1 |
| rs2568958 | 2,094 | 0.097 | 0.157 | 0.538 | 1 | 973 | 0.077 | 0.242 | 0.751 | 1 | 1,121 | 0.180 | 0.205 | 0.382 | 1 |
| rs2890652i | 2,098 | -0.087 | 0.216 | 0.686 | 1 | 976 | -0.170 | 0.379 | 0.654 | 1 | 1,122 | -0.100 | 0.256 | 0.696 | 1 |
| rs29941 | 2,095 | -0.051 | 0.156 | 0.745 | 1 | 976 | 0.179 | 0.244 | 0.463 | 1 | 1,119 | -0.234 | 0.204 | 0.252 | 1 |
| rs3810291i | 2,098 | -0.220 | 0.171 | 0.197 | 1 | 976 | -0.678 | 0.307 | 0.027 | 1 | 1,122 | -0.035 | 0.200 | 0.862 | 1 |
| rs4430796 | 2,086 | 0.021 | 0.163 | 0.898 | 1 | 973 | -0.159 | 0.273 | 0.559 | 1 | 1,113 | 0.127 | 0.202 | 0.530 | 1 |
| rs4712652 | 2,096 | 0.078 | 0.154 | 0.610 | 1 | 975 | 0.089 | 0.262 | 0.734 | 1 | 1,121 | 0.045 | 0.184 | 0.808 | 1 |
| rs4771122i | 2,098 | 0.280 | 0.188 | 0.137 | 1 | 976 | 0.278 | 0.320 | 0.386 | 1 | 1,122 | 0.198 | 0.224 | 0.378 | 1 |
| rs4929949i | 2,098 | -0.084 | 0.174 | 0.628 | 1 | 976 | 0.390 | 0.282 | 0.166 | 1 | 1,122 | -0.461 | 0.216 | 0.033 | 1 |
| rs543874i | 2,098 | 0.122 | 0.184 | 0.507 | 1 | 976 | -0.005 | 0.319 | 0.987 | 1 | 1,122 | 0.146 | 0.220 | 0.506 | 1 |
| rs560887 | 2,096 | 0.146 | 0.187 | 0.433 | 1 | 975 | -0.170 | 0.339 | 0.617 | 1 | 1,121 | 0.240 | 0.214 | 0.264 | 1 |
| rs6013029i | 2,098 | -0.350 | 0.423 | 0.408 | 1 | 976 | 0.123 | 0.594 | 0.836 | 1 | 1,122 | -1.139 | 0.625 | 0.068 | 1 |
| rs6232 | 2,096 | -0.725 | 0.338 | 0.032 | 1 | 975 | -0.594 | 0.714 | 0.405 | 1 | 1,121 | -0.614 | 0.363 | 0.091 | 1 |
| rs6602024i | 2,098 | 0.121 | 0.252 | 0.632 | 1 | 976 | -0.085 | 0.405 | 0.833 | 1 | 1,122 | 0.248 | 0.320 | 0.438 | 1 |
| rs713586i | 2,098 | 0.243 | 0.159 | 0.126 | 1 | 976 | 0.099 | 0.316 | 0.755 | 1 | 1,122 | 0.333 | 0.176 | 0.058 | 1 |
| rs7647305 | 2,097 | 0.349 | 0.216 | 0.106 | 1 | 976 | -0.019 | 0.359 | 0.958 | 1 | 1,121 | 0.655 | 0.262 | 0.012 | 0.462 |
| rs7961581i | 2,098 | -0.063 | 0.194 | 0.746 | 1 | 976 | -0.294 | 0.338 | 0.386 | 1 | 1,122 | 0.076 | 0.228 | 0.739 | 1 |
| rs9939609i | 2,098 | -0.021 | 0.178 | 0.908 | 1 | 976 | -0.275 | 0.320 | 0.39 | 1 | 1,122 | 0.019 | 0.210 | 0.927 | 1 |
| *^1^An "i" following the rs-number indicates that imputed SNP information was used.*  *^2^Calculated using linear regression. Adjusted for pre-pregnancy BMI, maternal age at conception, gestational age at birth, parity, social-occupational status, physical activity, smoking and alcohol intake during pregnancy.*  *^3^Bonferroni adjusted P-value* | | | | | | | | | | | | | | | |
