# Supplementary material for: Association between Maternal Fish Consumption and Gestational Weight Gain: Influence of Molecular Genetic Predisposition to Obesity
Source: PLoS One. 2016 Mar 1;11(3):e0150105. doi: 10.1371/journal.pone.0150105 (PMC4773113; doi:10.1371/journal.pone.0150105)
Supplement: S4 Table — (DOCX) [file pone.0150105.s006.docx]

| **S4 Table. SNP × fatty fish interaction in relation to gestational weight gain, presented in kg per additional risk allele for each serving of fatty fish** | | | | | | | | | | | | | | | |
| --- | --- | --- | --- | --- | --- | --- | --- | --- | --- | --- | --- | --- | --- | --- | --- |
|  | All | | | | | Obese | | | | | Non-obese | | | | |
| SNP | N | β^2^ | SE | P | P_B_^3^ | N | β | SE | P | P_B_ | N | β | SE | P | P_B_ |
| rs10146997 | 2,088 | 0.482 | 0.342 | 0.159 | 1 | 970 | -1.824 | 0.819 | 0.026 | 0.959 | 1,118 | 1.18 | 0.357 | 0.001 | 0.035 |
| rs10508503 | 2,087 | 0.131 | 0.475 | 0.782 | 1 | 969 | 1.385 | 1.384 | 0.317 | 1 | 1,118 | -0.204 | 0.501 | 0.684 | 1 |
| rs10838738 | 2,089 | -0.165 | 0.361 | 0.648 | 1 | 971 | 0.644 | 0.701 | 0.358 | 1 | 1,118 | -0.603 | 0.404 | 0.136 | 1 |
| rs10938397i^1^ | 2,091 | 0.110 | 0.360 | 0.759 | 1 | 971 | 0.174 | 0.743 | 0.815 | 1 | 1,120 | 0.114 | 0.394 | 0.773 | 1 |
| rs10968576 | 2,090 | -0.022 | 0.382 | 0.954 | 1 | 971 | -0.893 | 0.625 | 0.153 | 1 | 1,119 | 0.614 | 0.475 | 0.196 | 1 |
| rs1121980i | 2,091 | 0.262 | 0.390 | 0.502 | 1 | 971 | 0.096 | 0.764 | 0.900 | 1 | 1,120 | 0.343 | 0.438 | 0.433 | 1 |
| rs11847697i | 2,091 | 1.321 | 0.704 | 0.061 | 1 | 971 | 0.146 | 1.429 | 0.919 | 1 | 1,120 | 1.787 | 0.770 | 0.020 | 0.753 |
| rs12444979i | 2,091 | -0.213 | 0.570 | 0.708 | 1 | 971 | -1.234 | 1.100 | 0.262 | 1 | 1,120 | 0.054 | 0.641 | 0.933 | 1 |
| rs13107325 | 2,090 | -0.297 | 0.840 | 0.724 | 1 | 971 | 0.451 | 1.444 | 0.755 | 1 | 1,119 | -1.257 | 1.019 | 0.217 | 1 |
| rs1424233 | 2,088 | -0.699 | 0.302 | 0.021 | 0.765 | 970 | -0.399 | 0.552 | 0.470 | 1 | 1,118 | -0.849 | 0.344 | 0.013 | 0.498 |
| rs1514175 | 2,079 | -0.626 | 0.335 | 0.062 | 1 | 967 | -1.491 | 0.786 | 0.058 | 1 | 1,112 | -0.415 | 0.353 | 0.24 | 1 |
| rs1555543i | 2,091 | -0.686 | 0.441 | 0.120 | 1 | 971 | -0.587 | 0.724 | 0.417 | 1 | 1,120 | -0.753 | 0.544 | 0.166 | 1 |
| rs17782313i | 2,091 | -0.203 | 0.337 | 0.547 | 1 | 971 | -0.531 | 0.805 | 0.509 | 1 | 1,120 | -0.185 | 0.354 | 0.602 | 1 |
| rs1801282i | 2,091 | -1.597 | 0.578 | 0.006 | 0.211 | 971 | -1.838 | 0.993 | 0.064 | 1 | 1,120 | -1.671 | 0.691 | 0.016 | 0.577 |
| rs1805081 | 2,089 | 0.679 | 0.342 | 0.047 | 1 | 971 | 0.757 | 0.582 | 0.194 | 1 | 1,118 | 0.513 | 0.412 | 0.213 | 1 |
| rs206936i | 2,091 | -0.500 | 0.503 | 0.320 | 1 | 971 | -0.041 | 0.965 | 0.966 | 1 | 1,120 | -0.620 | 0.564 | 0.272 | 1 |
| rs2112347i | 2,091 | 0.091 | 0.371 | 0.806 | 1 | 971 | -0.535 | 0.633 | 0.398 | 1 | 1,120 | 0.360 | 0.460 | 0.435 | 1 |
| rs2237892 | 2,090 | 0.864 | 0.608 | 0.155 | 1 | 971 | 1.180 | 1.259 | 0.349 | 1 | 1,119 | 0.616 | 0.662 | 0.352 | 1 |
| rs2241423 | 2,089 | 1.425 | 0.449 | 0.001 | 0.055 | 971 | 3.134 | 0.907 | 0.001 | 0.02 | 1,118 | 0.806 | 0.500 | 0.107 | 1 |
| rs2287019 | 2,090 | -0.369 | 0.426 | 0.387 | 1 | 971 | -0.160 | 0.716 | 0.823 | 1 | 1,119 | -0.522 | 0.526 | 0.320 | 1 |
| rs2568958 | 2,087 | -0.035 | 0.381 | 0.927 | 1 | 968 | -0.284 | 0.630 | 0.652 | 1 | 1,119 | 0.128 | 0.465 | 0.783 | 1 |
| rs2890652i | 2,091 | 0.644 | 0.482 | 0.181 | 1 | 971 | -0.131 | 0.926 | 0.888 | 1 | 1,120 | 0.893 | 0.543 | 0.100 | 1 |
| rs29941 | 2,088 | 0.520 | 0.369 | 0.159 | 1 | 971 | 1.030 | 0.647 | 0.111 | 1 | 1,117 | 0.237 | 0.446 | 0.595 | 1 |
| rs3810291i | 2,091 | -0.230 | 0.355 | 0.517 | 1 | 971 | -1.896 | 0.644 | 0.003 | 0.119 | 1,120 | 0.590 | 0.409 | 0.149 | 1 |
| rs4430796 | 2,079 | 0.587 | 0.354 | 0.098 | 1 | 968 | 0.532 | 0.708 | 0.453 | 1 | 1,111 | 0.687 | 0.392 | 0.080 | 1 |
| rs4712652 | 2,089 | 0.277 | 0.329 | 0.399 | 1 | 970 | -0.346 | 0.554 | 0.532 | 1 | 1,119 | 0.69 | 0.397 | 0.082 | 1 |
| rs4771122i | 2,091 | 0.786 | 0.440 | 0.074 | 1 | 971 | 0.800 | 0.800 | 0.317 | 1 | 1,120 | 0.745 | 0.510 | 0.144 | 1 |
| rs4929949i | 2,091 | -0.103 | 0.389 | 0.792 | 1 | 971 | 0.997 | 0.658 | 0.13 | 1 | 1,120 | -0.855 | 0.467 | 0.067 | 1 |
| rs543874i | 2,091 | 0.532 | 0.394 | 0.178 | 1 | 971 | 0.643 | 0.756 | 0.394 | 1 | 1,120 | 0.382 | 0.444 | 0.390 | 1 |
| rs560887 | 2,089 | -0.133 | 0.403 | 0.742 | 1 | 970 | -0.396 | 0.793 | 0.618 | 1 | 1,119 | -0.190 | 0.446 | 0.671 | 1 |
| rs6013029i | 2,091 | 0.074 | 0.849 | 0.931 | 1 | 971 | 0.371 | 1.183 | 0.754 | 1 | 1,120 | -0.238 | 1.318 | 0.857 | 1 |
| rs6232 | 2,089 | -1.062 | 0.849 | 0.211 | 1 | 970 | -0.558 | 1.393 | 0.689 | 1 | 1,119 | -1.202 | 1.050 | 0.252 | 1 |
| rs6602024i | 2,091 | 0.340 | 0.632 | 0.591 | 1 | 971 | -0.257 | 1.185 | 0.828 | 1 | 1,120 | 0.611 | 0.720 | 0.396 | 1 |
| rs713586i | 2,091 | 0.520 | 0.365 | 0.154 | 1 | 971 | 0.182 | 0.759 | 0.810 | 1 | 1,120 | 0.687 | 0.396 | 0.083 | 1 |
| rs7647305 | 2,090 | 0.689 | 0.474 | 0.146 | 1 | 971 | -0.065 | 0.826 | 0.937 | 1 | 1,119 | 1.290 | 0.568 | 0.023 | 0.856 |
| rs7961581i | 2,091 | 0.367 | 0.416 | 0.378 | 1 | 971 | -0.736 | 0.894 | 0.411 | 1 | 1,120 | 0.902 | 0.447 | 0.043 | 1 |
| rs9939609i | 2,091 | -0.332 | 0.369 | 0.369 | 1 | 971 | -0.001 | 0.797 | 0.999 | 1 | 1,120 | -0.523 | 0.403 | 0.195 | 1 |
| *^1^An "i" following the rs-number indicates that imputed SNP information was used.*  *^2^Calculated using linear regression. Adjusted for pre-pregnancy BMI, maternal age at conception, gestational age at birth, parity, social-occupational status, physical activity, smoking and alcohol intake during pregnancy.*  *^3^Bonferroni adjusted P-value* | | | | | | | | | | | | | | | |
