# Supplementary material for: Association between Maternal Fish Consumption and Gestational Weight Gain: Influence of Molecular Genetic Predisposition to Obesity
Source: PLoS One. 2016 Mar 1;11(3):e0150105. doi: 10.1371/journal.pone.0150105 (PMC4773113; doi:10.1371/journal.pone.0150105)
Supplement: S5 Table — (DOCX) [file pone.0150105.s007.docx]

| **S5 Table. SNP × lean fish interaction in relation to gestational weight**  **gain , presented in kg per additional risk allele for each serving of lean fish** | | | | | | | | | | | | | | | |
| --- | --- | --- | --- | --- | --- | --- | --- | --- | --- | --- | --- | --- | --- | --- | --- |
|  | All | | | | | Obese | | | | | Non-obese | | | | |
| SNP | N | β^2^ | SE | P | P_B_^3^ | N | β | SE | P | P_B_ | N | β | SE | P | P_B_ |
| rs10146997 | 2,095 | 0.189 | 0.212 | 0.371 | 1 | 975 | -0.694 | 0.42 | 0.098 | 1 | 1,120 | 0.601 | 0.232 | 0.009 | 0.35 |
| rs10508503 | 2,094 | 0.067 | 0.312 | 0.829 | 1 | 974 | 0.675 | 0.636 | 0.289 | 1 | 1,120 | -0.227 | 0.349 | 0.516 | 1 |
| rs10838738 | 2,096 | -0.305 | 0.224 | 0.172 | 1 | 976 | 0.021 | 0.401 | 0.959 | 1 | 1,120 | -0.459 | 0.259 | 0.076 | 1 |
| rs10938397i^1^ | 2,098 | 0.007 | 0.224 | 0.975 | 1 | 976 | 0.857 | 0.399 | 0.032 | 1 | 1,122 | -0.490 | 0.263 | 0.062 | 1 |
| rs10968576 | 2,097 | 0.081 | 0.221 | 0.713 | 1 | 976 | -0.212 | 0.383 | 0.579 | 1 | 1,121 | 0.278 | 0.260 | 0.286 | 1 |
| rs1121980i | 2,098 | -0.067 | 0.241 | 0.780 | 1 | 976 | -0.374 | 0.412 | 0.363 | 1 | 1,122 | 0.072 | 0.293 | 0.807 | 1 |
| rs11847697i | 2,098 | 0.991 | 0.455 | 0.029 | 1 | 976 | -0.978 | 1.042 | 0.348 | 1 | 1,122 | 1.646 | 0.481 | 0.001 | 0.023 |
| rs12444979i | 2,098 | -0.133 | 0.295 | 0.651 | 1 | 976 | -0.491 | 0.519 | 0.345 | 1 | 1,122 | 0.031 | 0.347 | 0.928 | 1 |
| rs13107325 | 2,097 | 0.306 | 0.544 | 0.573 | 1 | 976 | 0.447 | 1.030 | 0.665 | 1 | 1,121 | -0.076 | 0.621 | 0.903 | 1 |
| rs1424233 | 2,095 | -0.321 | 0.190 | 0.092 | 1 | 975 | 0.054 | 0.329 | 0.869 | 1 | 1,120 | -0.526 | 0.231 | 0.023 | 1 |
| rs1514175 | 2,086 | -0.045 | 0.197 | 0.819 | 1 | 972 | 0.153 | 0.320 | 0.632 | 1 | 1,114 | -0.225 | 0.262 | 0.39 | 1 |
| rs1555543i | 2,098 | 0.144 | 0.227 | 0.524 | 1 | 976 | 0.146 | 0.405 | 0.718 | 1 | 1,122 | 0.132 | 0.268 | 0.622 | 1 |
| rs17782313i | 2,098 | -0.338 | 0.236 | 0.151 | 1 | 976 | -0.414 | 0.393 | 0.293 | 1 | 1,122 | -0.349 | 0.287 | 0.224 | 1 |
| rs1801282i | 2,098 | -1.114 | 0.300 | 0.0002 | 0.007 | 976 | -0.539 | 0.539 | 0.317 | 1 | 1,122 | -1.572 | 0.347 | 0.00001 | 0.0002 |
| rs1805081 | 2,096 | 0.458 | 0.217 | 0.035 | 1 | 976 | 0.017 | 0.393 | 0.965 | 1 | 1,120 | 0.598 | 0.253 | 0.018 | 0.667 |
| rs206936i | 2,098 | -0.249 | 0.294 | 0.397 | 1 | 976 | -0.317 | 0.514 | 0.538 | 1 | 1,122 | -0.159 | 0.347 | 0.647 | 1 |
| rs2112347i | 2,098 | 0.067 | 0.199 | 0.738 | 1 | 976 | -0.106 | 0.319 | 0.738 | 1 | 1,122 | 0.174 | 0.258 | 0.501 | 1 |
| rs2237892 | 2,097 | 0.292 | 0.489 | 0.550 | 1 | 976 | 1.427 | 0.921 | 0.122 | 1 | 1,121 | -0.483 | 0.557 | 0.385 | 1 |
| rs2241423 | 2,096 | 0.405 | 0.252 | 0.108 | 1 | 976 | 0.420 | 0.450 | 0.351 | 1 | 1,120 | 0.433 | 0.294 | 0.141 | 1 |
| rs2287019 | 2,097 | -0.092 | 0.271 | 0.735 | 1 | 976 | 0.163 | 0.473 | 0.730 | 1 | 1,121 | -0.184 | 0.319 | 0.564 | 1 |
| rs2568958 | 2,094 | 0.096 | 0.206 | 0.642 | 1 | 973 | 0.194 | 0.317 | 0.540 | 1 | 1,121 | 0.058 | 0.275 | 0.834 | 1 |
| rs2890652i | 2,098 | 0.127 | 0.274 | 0.641 | 1 | 976 | -0.234 | 0.495 | 0.636 | 1 | 1,122 | 0.288 | 0.323 | 0.373 | 1 |
| rs29941 | 2,095 | 0.026 | 0.202 | 0.899 | 1 | 976 | 0.038 | 0.314 | 0.903 | 1 | 1,119 | 0.027 | 0.268 | 0.918 | 1 |
| rs3810291i | 2,098 | -0.308 | 0.251 | 0.221 | 1 | 976 | -0.414 | 0.437 | 0.344 | 1 | 1,122 | -0.291 | 0.301 | 0.332 | 1 |
| rs4430796 | 2,086 | 0.191 | 0.207 | 0.356 | 1 | 973 | -0.410 | 0.354 | 0.248 | 1 | 1,113 | 0.595 | 0.249 | 0.017 | 0.616 |
| rs4712652 | 2,096 | 0.316 | 0.215 | 0.141 | 1 | 975 | 0.321 | 0.383 | 0.402 | 1 | 1,121 | 0.265 | 0.252 | 0.292 | 1 |
| rs4771122i | 2,098 | 0.518 | 0.249 | 0.038 | 1 | 976 | 0.332 | 0.432 | 0.442 | 1 | 1,122 | 0.542 | 0.293 | 0.064 | 1 |
| rs4929949i | 2,098 | -0.127 | 0.236 | 0.589 | 1 | 976 | 0.352 | 0.389 | 0.365 | 1 | 1,122 | -0.485 | 0.288 | 0.093 | 1 |
| rs543874i | 2,098 | 0.336 | 0.252 | 0.182 | 1 | 976 | -0.271 | 0.426 | 0.525 | 1 | 1,122 | 0.733 | 0.307 | 0.017 | 0.632 |
| rs560887 | 2,096 | -0.044 | 0.248 | 0.860 | 1 | 975 | -0.121 | 0.447 | 0.786 | 1 | 1,121 | -0.082 | 0.287 | 0.774 | 1 |
| rs6013029i | 2,098 | -1.100 | 0.632 | 0.082 | 1 | 976 | -0.015 | 0.926 | 0.987 | 1 | 1,122 | -2.376 | 0.868 | 0.006 | 0.229 |
| rs6232 | 2,096 | -1.181 | 0.457 | 0.010 | 0.363 | 975 | -0.928 | 0.945 | 0.326 | 1 | 1,121 | -1.112 | 0.495 | 0.025 | 0.917 |
| rs6602024i | 2,098 | -0.084 | 0.321 | 0.794 | 1 | 976 | -0.044 | 0.500 | 0.929 | 1 | 1,122 | -0.147 | 0.430 | 0.733 | 1 |
| rs713586i | 2,098 | 0.369 | 0.223 | 0.097 | 1 | 976 | 0.165 | 0.421 | 0.695 | 1 | 1,122 | 0.510 | 0.251 | 0.042 | 1 |
| rs7647305 | 2,097 | 0.587 | 0.288 | 0.042 | 1 | 976 | -0.008 | 0.485 | 0.987 | 1 | 1,121 | 1.044 | 0.347 | 0.003 | 0.097 |
| rs7961581i | 2,098 | 0.070 | 0.259 | 0.788 | 1 | 976 | -0.312 | 0.421 | 0.459 | 1 | 1,122 | 0.313 | 0.318 | 0.325 | 1 |
| rs9939609i | 2,098 | -0.324 | 0.231 | 0.160 | 1 | 976 | -0.405 | 0.412 | 0.326 | 1 | 1,122 | -0.425 | 0.281 | 0.130 | 1 |
| *^1^An "i" following the rs-number indicates that imputed SNP information was used.*  *^2^Calculated using linear regression. Adjusted for pre-pregnancy BMI, maternal age at conception, gestational age at birth, parity, social-occupational status, physical activity, smoking and alcohol intake during pregnancy.*  *^3^Bonferroni adjusted P-value* | | | | | | | | | | | | | | | |
